# Dr. Donné on the Means by Which We May Detect the Fact of Masturbation in Young Children

**Published:** 1842-11-26

**Authors:** 


### ANALECTA.

Dr. Donné, in his late work, "*Conseils aux mères*," indicates the means by which we may detect, by microscopical examination, the fact of masturbation in young children, without exciting any suspicion on their part, and avoid the necessity of personal inquiries. The urine of children addicted to this vice, contains, even before the age of puberty, certain matters which observation reveals to us, when care is taken to obtain it a short time after the child has abandoned himself to this fatal habit. This fact can be ascertained, not, as will be readily imagined, by the presence of spermatic animalcules, but by the existence of a certain quantity of mucus, coming either from the prostate or from the vesiculæ seminales, mixed with chrystals of the oxalate of lime in greater or less quantity. The presence of this substance in urine, which contains some one of the elements of the semen, is so constant a phenomenon that by it we can diagnosticate seminal emissions in the male, when the essential signs are wanting. This fact is seen in children. Attention, however, must be paid to the diet at the time; the use of sorrel, for example, is capable of producing in every one the formation of similar chrystals in the urine; but in this case it is an accidental phenomenon, and one which it is easy to take into account.

